# Factors Associated with Hispanic Adults Attending Spanish-Language Disease Self-Management Program Workshops and Workshop Completion

**DOI:** 10.3389/fpubh.2014.00155

**Published:** 2015-04-27

**Authors:** Matthew Lee Smith, SangNam Ahn, Luohua Jiang, Kristie P. Kulinski, Marcia G. Ory

**Affiliations:** ^1^Department of Health Promotion and Behavior, College of Public Health, The University of Georgia, Athens, GA, USA; ^2^Division of Health Systems Management and Policy, School of Public Health, The University of Memphis, Memphis, TN, USA; ^3^Department of Health Promotion and Community Health Sciences, Texas A&M Health Science Center, School of Public Health, College Station, TX, USA; ^4^Department of Epidemiology and Biostatistics, Texas A&M Health Science Center, School of Public Health, College Station, TX, USA; ^5^National Council on Aging, Washington, DC, USA

**Keywords:** chronic disease self-management, evidence-based program, Hispanic adults, intervention dose, Spanish language

## Abstract

Many factors influence ways in which middle-aged and older Hispanic adults prefer to receive health-related information. While Spanish-language disease management programs are increasingly offered in community and healthcare settings, less is known about their utilization among the Hispanic population. This study aimed to identify participant and workshop factors associated with middle-aged and older Hispanic adults attending Spanish-language disease self-management program workshops and receiving the recommended intervention dose (i.e., successful workshop completion is defined as attending four or more of the six workshop sessions). Data were analyzed from 12,208 Hispanic adults collected during a national dissemination of the Stanford suite of Chronic Disease Self-Management Education (CDSME) programs spanning 45 states, the District of Columbia, and Puerto Rico. Two logistic regression analyses were performed. Over 65% of participants attended Spanish-language workshops, and 78.3% of participants successfully completed workshops. Relative to participants in English-language workshops, participants who attended Spanish-language CDSME workshops were more likely to successfully complete workshops, as were those aged 80 years and older, females, and those who lived alone. Participants who were aged 50–79 years and female were significantly more likely to attend Spanish-language workshops than their counterparts under age 50. Conversely, those with more chronic conditions were less likely to attend Spanish-language workshops. Those who attended workshops with more participants and where the Hispanic population was less affluent were more likely to attend Spanish-language workshops. This study provides insight into Spanish-language CDSME program recruitment and utilization with implications for program adoption in underserved Hispanic community settings.

## Introduction

The United States is becoming increasingly more racially and ethnically diverse (1). The Hispanic population is the largest and fastest growing minority group in the United States (2). This population is expected to represent nearly one-third of the American population and one-fifth of the older adult population by 2050 (3). Growth rates are anticipated to be even higher in some parts of America such as the Texas–Mexico border (4).

The pattern of chronic disease differs among minority groups, and Hispanic individuals often acquire chronic conditions at younger ages than their non-Hispanic white counterparts (5). Additionally, as a group, Hispanic individuals are disproportionately burdened by chronic conditions including obesity, diabetes, and heart disease (6–9). They are also less likely to have access to health care (10, 11) or evidence-based health promotion programs (12). Despite the growing availability of evidence-based disease prevention programs for seniors (13, 14), language and/or cultural barriers may prevent Hispanic individuals from accessing these services (10).

English or Spanish-language preferences for receiving health information and materials among Hispanic individuals vary by a multitude of factors (15–17), but less is known about language-based preferences for evidence-based programs among this population. As such, this study draws from national data to examine participant and workshop characteristics associated with Hispanic individuals’ attending Spanish-language disease self-management program workshops. Further, this study examines if this participant subgroup received the recommended intervention dose (i.e., successfully completed the workshop by attending four or more of the six workshop sessions).

## Materials and Methods

### Program description

The Chronic Disease Self-Management Program (CDSMP) is one intervention in a suite of Chronic Disease Self-Management Education (CDSME) programs licensed through the Stanford Patient Education Research Center. CDSMP has been introduced and widely disseminated in the U.S. as a method to empower patients with self-management skills to deal with their chronic conditions (18). CDSMP is an evidence-based, peer-led intervention consisting of six highly participative classes held for 2.5 h each, once a week, for consecutive 6 weeks. (18) CDSMP has resulted in improved health care and health (19, 20), while potentially saving healthcare costs (21). While some of the CDSME programs are general (e.g., CDSMP), others are disease specific (e.g., diabetes, arthritis, chronic pain). While the chronic condition may vary, all CDSME programs are based upon social learning theory (22), highly interactive, and apply the principles of goal setting, problem solving, and action planning (22).

### Data source and study population

Cross-sectional data for this study were obtained from a nationwide delivery of CDSME programs as part of the American Recovery and Reinvestment Act of 2009 (i.e., ARRA) *Communities Putting Prevention to Work: Chronic Disease Self-Management Program* initiative (13, 23). The U.S. Administration on Aging led this initiative in collaboration with the Centers for Disease Control and Prevention and the Centers for Medicare and Medicaid Services to support the translation of CDSME programs in 45 states, Puerto Rico, and the District of Columbia (24). For this study, cases were only drawn from Hispanic participants within the first 100,000 participants enrolled in CDSME program workshops and who had complete data on variables of interest. Based on these inclusion criteria, the final analytic sample was 12,208 middle-aged and older Hispanic adults who attended a CDSMP workshop.

### Measures

#### Dependent variables

Two dependent variables were used for this study. Participants’ attendance was recorded to determine if the recommended intervention dose was received. As defined by the program developers, a participant has “successfully” completed the program if they attended four or more of the six offered workshop sessions (13, 19, 20, 25). Therefore, successful program completion was used as the first dependent variable in this study (i.e., non-successful completion served as the referent group). The second dependent variable was the workshop language in which attended. Workshops are offered in approximately 20 languages worldwide (25). Although CDSME program workshops are available in a variety of languages other than English (e.g., Mandarin Chinese, Korean, Farsi, Tagalog), the most predominant non-English workshop language is Spanish. Therefore, participants’ enrollment in Spanish-language workshops was used as the second dependent variable in this study (i.e., enrollment in English-language workshops served as the referent group). Spanish-language CDSME programs offered in this nationwide rollout included *Tomando Control de su Salud* (i.e., Spanish version of CDSMP), *Programa de Manejo Personal de la Diabetes* (i.e., Spanish version of the diabetes self-management program), and *Curso de Manejo Personal de la Artritis* (i.e., Spanish version of the arthritis self-management program).

#### Personal characteristics

Personal characteristics of the participants included age group (i.e., under 50 years, 50–64 years, 65–79 years, 80+ years), sex (i.e., male, female), living situation (i.e., lives alone, lives with others), and self-reported number of chronic conditions (i.e., ranging from 0 to 10). Chronic condition types included arthritis, cancer, depression, diabetes, heart disease, hypertension, lung disease, stroke, osteoporosis, and other chronic conditions.

#### Delivery site types

Data pertaining to CDSME program delivery site types were gathered administratively, as described previously. Delivery site types included healthcare organizations, senior centers or area agencies on aging (AAAs), residential facilities, community or multi-purpose centers (including libraries), faith-based organizations, educational institutions, and site types classified as “other” (e.g., correctional facilities malls, RV parks, fire departments, county administration buildings, private residences, casinos, career centers).

#### Neighborhood characteristics

Using participants’ residential ZIP Codes, geographic information system (GIS) software was used to generate neighborhood-level variables for each participant. Neighborhood characteristics included residential rurality (i.e., metro residence or non-metro residence based on the rural–urban commuting area codes (RUCA) (26)) and the percent of Hispanic families below the federal poverty line residing in the participants’ ZIP Code (27). Using organizational ZIP Codes, GIS software was used to generate neighborhood-level variables for each delivery site (i.e., site rurality, percent of Hispanic families below the federal poverty line).

### Analyses

All statistical analyses were performed using SPSS (version 21). Of the first 100,000 participants reached in this initiative, all non-Hispanic cases (*n* = 86,191) were immediately omitted from analyses based on specified study aims, which left 13,809 Hispanic participants. Of these Hispanic participants, those with missing data for age (*n* = 661), sex (*n* = 291), living situation (*n* = 8), residential rurality (*n* = 973), delivery site rurality (*n* = 11), and class size (*n* = 85) were omitted. Some participants had more than one of these exclusionary characteristics, thus the final sample was 12,208 middle-aged and older Hispanic adults who attended a CDSMP workshop. When comparing characteristics between Hispanic participants in the analytic sample with Hispanic participants omitted from analyses, participants in the analytic sample were significantly younger, lived with others, and had more chronic conditions. No significant differences were observed based on participants’ sex or the rurality of their residence.

For participants meeting study inclusion criteria, frequencies were calculated for all major study variables, which were initially examined in relationship to participants’ successful workshop completion and the workshop language in which participants attended. Pearson’s chi-square tests were performed to assess differences between categorical independent variables. Independent sample *t*-tests were used to examine mean differences for continuous variables. Two logistic regression analyses were performed to identify factors associated with attending Spanish-language workshops (i.e., attending English-language workshops served as the referent group) and successful workshop attendance (i.e., non-successful attendance served as the referent group). Odds ratios and 95% confidence intervals are reported.

## Results

### Sample characteristics

Sample characteristics of study participants are presented in Table 1. Of the 12,208 study participants, 65.1% attended Spanish-language workshops and 78.3% successfully completed the program (i.e., attended four or more of the six offered workshop sessions). Over 55% of participants were aged 64 years or younger and 78.4% was female. On average, participants self-reported 1.96 (±1.54) chronic conditions. The majority of participants lived with others (92.2%) and resided in metro areas (93.2%). The largest proportion of these Hispanic participants attended workshops at healthcare organizations (32.1%), followed by senior centers or AAAs (22.3%), residential facilities (11.1%), and community or multi-purpose centers (10.1%). On average, workshops had 13.14 (±4.08) participants, and participants attended 4.49 (±1.64) of the six workshop sessions.

**Table 1:** **Sample characteristics by workshop language and completion**.

	Total (*n* = 12,208)	Workshop language				Workshop completion			
		English (*n* = 4,262)	Spanish (*n* = 7,946)	χ^2^ or *t*	*P*	Not successful (*n* = 2,652)	Successful (*n* = 9,556)	χ^2^ or *t*	*P*
Work shop completion				58.52	<0.001				
Not successful	21.7%	25.6%	19.6%			–	–	–	–
Successful	78.3%	74.4%	80.4%			–	–	–	–
Work shop language								58.52	<0.001
English	34.9%	–	–	–	–	41.2%	33.2%		
Spanish	65.1%	–	–	–	–	58.8%	66.8%		
Age				149.44	<0.001			26.16	<0.001
Under 50	25.6%	19.5%	28.9%			26.9%	25.2%		
50–64	29.8%	30.0%	29.7%			31.3%	29.4%		
65–79	34.8%	39.6%	32.2%			30.8%	35.9%		
80+	9.8%	11.0%	9.1%			11.0%	9.4%		
Sex				59.47	<0.001			22.42	<0.001
Male	21.6%	25.6%	19.5%			25.0%	20.7%		
Female	78.4%	74.4%	80.5%			75.0%	79.3%		
Living situation				14.09	<0.001			20.41	<0.001
Lives with others	92.2%	93.4%	91.5%			94.3%	91.6%		
Lives alone	7.8%	6.6%	8.5%			5.7%	8.4%		
Rurality (participant residence)				374.95	<0.001			4.72	0.030
Metro	93.2%	87.2%	96.4%			92.3%	93.5%		
Non-metro	6.8%	12.8%	3.6%			7.7%	6.5%		
Number of chronic conditions	1.96 (±1.54)	2.24 (±1.62)	1.81 (±1.47)	14.36	<0.001	1.96 (±1.57)	1.96 (±1.53)	−0.22	0.825
Percent of Hispanics below poverty (participant residence)	10.41 (±10.26)	6.30 (±5.66)	12.61 (±11.43)	−40.79	<0.001	9.59 (±8.96)	10.63 (±10.58)	−5.10	<0.001
Delivery site type				464.88	<0.001			86.17	<0.001
Healthcare Organization	32.1%	24.7%	36.0%			38.2%	30.4%		
Senior center/AAA	22.3%	29.5%	18.4%			20.1%	22.9%		
Residential facility	11.1%	13.2%	10.0%			12.1%	10.9%		
Community/Multi-Purpose Center	10.1%	10.1%	10.2%			10.2%	10.1%		
Faith-based Organization	6.6%	5.1%	7.5%			5.6%	6.9%		
Educational Institution	4.1%	1.3%	5.6%			3.5%	4.2%		
Other	13.6%	16.0%	12.3%			10.3%	14.5%		
Rurality (delivery site location)				390.99	<0.001			0.38	0.537
Metro	93.7%	87.7%	96.9%			93.4%	93.7%		
Non-metro	6.3%	12.3%	3.1%			6.6%	6.3%		
Class size	13.14 (±4.08)	12.60 (±4.16)	13.43 (±4.00)	−10.61	<0.001	13.35 (±4.06)	13.08 (±4.08)	3.09	0.002
Number of sessions attended	4.49 (±1.64)	4.33 (±1.70)	4.57 (±1.61)	−7.56	<0.001	1.76 (±0.82)	5.25 (±0.79)	–195.38	<0.001
Percent of Hispanics below poverty (delivery site location)	10.51 (±10.25)	6.35 (±5.64)	12.73 (±11.42)	−41.30	<0.001	9.80 (±9.20)	10.70 (±10.52)	−4.34	<0.001

### Attending Spanish-language workshops

Significant differences were observed when comparing sample characteristics by workshop language in bivariate analyses (see Table 1). A significantly larger proportion of participants who enrolled in Spanish-language workshops also received the recommended intervention dose (i.e., attended four or more of the six workshop sessions) (χ^2^ = 58.52, *P* < 0.001). Significantly larger proportions of younger participants (χ^2^ = 149.44, *P* < 0.001) and female participants (χ^2^ = 59.47, *P* < 0.001) attended Spanish-language workshops. On average, participants who attended Spanish-language workshops had fewer chronic conditions (*t* = 14.36, *P* < 0.001). Significantly larger proportions of participants who lived alone (χ^2^ = 14.09, *P* < 0.001) and lived in metro areas (χ^2^ = 374.95, *P* < 0.001) attended Spanish-language workshops. On average, those attending Spanish-language workshops resided (*t* = −40.79, *P* < 0.001) and attended delivery sites (*t* = −41.30, *P* < 0.001) in areas with higher percentages of Hispanic families below the federal poverty line. Larger proportions of participants who attended Spanish-language workshops did so at healthcare organizations and educational institutions, whereas smaller proportions of participants who attended Spanish-language workshops did so at senior centers or AAAs and residential facilities (χ^2^ = 464.88, *P* < 0.001). On average, participants who attended Spanish-language workshops had larger class sizes (*t* = −10.61, *P* < 0.001) and attended more workshop sessions (*t* = −7.56, *P* < 0.001).

Table 2 presents the logistic regression modeling factors associated with Hispanic participants’ enrollment in Spanish-language workshops (i.e., attending English-language workshops served as the referent group). Compared to participants under age 50 years, those who were aged 50–64 years (OR = 1.68, *P* < 0.001) and 65–79 years (OR = 1.29, *P* = 0.002) were significantly more likely to attend Spanish-language workshops. Female participants were also more likely to attend Spanish-language workshops (OR = 1.26, *P* < 0.001), whereas, those with fewer chronic conditions (OR = 0.85, *P* < 0.001) and who resided in non-metro areas (OR = 0.28, *P* < 0.001) were less likely to attend Spanish-language workshops. Relative to those who attended workshops in healthcare organizations, participants who attended workshops at all other delivery site types, except residential facilities, were significantly more likely to attend Spanish-language workshops (*P* < 0.001). Participants in workshops with more participants (OR = 1.03, *P* < 0.001) and those attending workshops at delivery sites in areas with higher percentages of Hispanic families below the federal poverty line (OR = 1.15, *P* < 0.001) were significantly more likely to attend Spanish-language workshops.

**Table 2:** **Factors associated with enrollment in Spanish-language workshops**.

	OR	*P*	95% CI	
			Lower	Upper
Age: under 50	1.00	–	–	–
Age: 50–64	1.68	<0.001	1.41	1.99
Age: 65–79	1.29	0.002	1.10	1.51
Age: 80+	0.97	0.681	0.83	1.13
Male	1.00	–	–	–
Female	1.26	<0.001	1.15	1.40
Lives with others	1.00	–	–	–
lives alone	1.12	0.179	0.95	1.33
Metro (participant-level)	1.00	–	–	–
Non-metro (participant-level)	0.28	<0.001	0.21	0.39
Number of chronic conditions	0.85	<0.001	0.82	0.87
Percent of Hispanics below poverty (participant-level)	0.98	0.287	0.95	1.02
Delivery site: Healthcare Organization	1.00	–	–	–
Delivery site: senior center/AAA	2.27	<0.001	1.98	2.60
Delivery site: residential facility	1.07	0.400	0.92	1.24
Delivery site: Community/Multi-Purpose Center	1.60	<0.001	1.35	1.90
Delivery site: Faith-Based Organization	1.96	<0.001	1.64	2.34
Delivery site: Educational Institution	3.46	<0.001	2.82	4.24
Delivery site: other	5.14	<0.001	3.72	7.11
Metro (delivery site-level)	1.00	–	–	–
Non-metro (delivery site-level)	0.74	0.072	0.53	1.03
Class size	1.03	<0.001	1.02	1.04
Percent of Hispanics below poverty (delivery site-level)	1.15	<0.001	1.11	1.19

### Successful workshop completion

Significant differences were observed when comparing sample characteristics by workshop completion in bivariate analyses (see Table 1). A significantly larger proportion of participants aged 65–79 years (χ^2^ = 26.16, *P* < 0.001) and female participants (χ^2^ = 22.42, *P* < 0.001) successfully completed workshops. Significantly larger proportions of participants who lived alone (χ^2^ = 20.41, *P* < 0.001) and lived in metro areas (χ^2^ = 4.72, *P* = 0.030) successfully completed workshops. On average, those who successfully completed workshops resided in (*t* = −5.10, *P* < 0.001) and attended delivery sites in (*t* = −4.34, *P* < 0.001) areas with higher percentages of Hispanic families below the federal poverty line. Larger proportions of participants who successfully completed workshops did so at senior centers or AAAs and other delivery sites, whereas smaller proportions of participants who successfully completed workshops did so at healthcare facilities and residential facilities (χ^2^ = 86.17, *P* < 0.001). On average, participants who successfully completed workshops were in workshops with fewer participants (*t* = 3.09, *P* = 0.002).

Table 3 presents the logistic regression modeling factors associated with successful workshop completion (i.e., attending fewer than four workshops served as the referent group). Compared to participants under age 50 years, those who were aged 85 years and older were significantly more likely to be successful completers (OR = 1.36, *P* < 0.001). Female participants (OR = 1.22, *P* < 0.001), those who lived alone (OR = 1.34, *P* = 0.002), and those who resided in areas with higher percentages of Hispanic families below the federal poverty line (OR = 1.05, *P* = 0.002) were more likely to successfully complete workshops. Relative to those who attended workshops in healthcare organizations, participants who attended workshops at all other delivery site types, except educational institutions, were significantly less likely to successfully complete workshops (*P* < 0.05). Participants enrolled in Spanish-language workshops were significantly more likely to successfully complete workshops (OR = 1.50, *P* < 0.001), whereas, those in workshops with larger class sizes (OR = 0.98, *P* < 0.001) were significantly less likely to successfully complete workshops. Those attending workshops at delivery sites in areas with higher percentages of Hispanic families below the federal poverty line (OR = 0.96, *P* = 0.004) were also significantly less likely to successfully complete workshops.

**Table 3:** **Factors associated with successful workshop completion**.

	OR	*P*	95% CI	
			Lower	Upper
Age: under 50	1.00	–	–	–
Age: 50–64	1.09	0.320	0.92	1.30
Age: 65–79	1.14	0.103	0.97	1.34
Age: 80+	1.36	<0.001	1.17	1.59
Male	1.00	–	–	–
Female	1.22	<0.001	1.10	1.35
Lives with others	1.00	–	–	–
Lives alone	1.34	0.002	1.11	1.61
Metro (participant-level)	1.00	–	–	–
Non-metro (participant-level)	0.81	0.210	0.57	1.13
Number of chronic conditions	1.01	0.530	0.98	1.04
Percent of Hispanics below poverty (participant-level)	1.05	0.002	1.02	1.08
Workshop: English	1.00	–	–	–
Workshop: Spanish	1.50	<0.001	1.36	1.66
Delivery site: Healthcare Organization	1.00	–	–	–
Delivery site: senior center/AAA	0.52	<0.001	0.45	0.61
Delivery site: residential facility	0.74	0.001	0.63	0.88
Delivery site: Community/Multi-Purpose Center	0.60	<0.001	0.50	0.73
Delivery site: Faith-based Organization	0.64	<0.001	0.53	0.78
Delivery site: Educational Institution	0.81	0.060	0.64	1.01
Delivery site: other	0.71	0.013	0.55	0.93
Metro (delivery site-level)	1.00	–	–	–
Non-metro (delivery site-level)	1.19	0.341	0.83	1.69
Class size	0.98	<0.001	0.97	0.99
Percent of Hispanics below poverty (delivery site-level)	0.96	0.004	0.93	0.99

## Discussion

Hispanic participants represented 17.4% of the first 100,000 participants reached through this ARRA implementation effort (28), a percentage that is representative of the overall Hispanic population in the United States (29). However, relative to the larger population reached in this initiative, participants in our sample are younger (25.6% under age 50 compared to 12.0% in the larger group) (28). This finding is important because it reinforces that Hispanics in the United States are acquiring chronic conditions at younger ages and living with those conditions for longer periods of time (5), thus highlighting the necessity for self-management programs. With approximately two-thirds of sample participants (*n* = 7,946) attending Spanish-language CDSME program workshops, this study supports previous studies’ assumptions about preferences among Hispanic individuals for receiving health-related information delivered in Spanish (15–17). While the proportion of Hispanic participants electing to attend Spanish-language workshops is substantial, further inspection of the larger initiative (28) reveals that the majority of workshops delivered to the first 100,000 participants were English-language CDSMP (78.4%) and the Diabetes Self-Management Program (10.3%), whereas only about 10% were specialized Spanish versions of the CDSME (i.e., Tomando Control de su Salud, Programa de Manejo Personal de la Diabetes, and Curso de Manejo Personal de la Artritis). Therefore, it remains to be determined if the number of Hispanic participants would have been larger if more Spanish-language workshops were available across the country, or if older Hispanics are becoming increasingly assimilated and comfortable with English for health-related information.

The overall completion rates among Hispanic participants were higher than for the total population of CDSME program participants (28) (i.e., 74.9% completion among all participants, 78.3% completion among Hispanic participants, and 80.4% completion among Spanish-language workshop participants), and can be attributed, in part, to the availability of Spanish-language workshops. This study identified participant and workshop characteristics associated with attendance at Spanish speaking versus English-language workshops. Relative to English-language workshops, Spanish-language workshops attracted a different population base (e.g., younger, female, fewer chronic conditions) and were held in different settings (e.g., more urban, less affluent settings). These differences may be attributed to other characteristics associated with successful Spanish-language workshop completion such as attending more workshops in healthcare facilities and senior centers/AAAs or attending workshops with larger class sizes.

Several research and practice implications emerge from this study. First, in future research, it will be important to stratify Hispanic participants by ethnic origin to identify characteristics contributing to their program enrollment, attendance, and benefits. As indicated in new census designations (30), there is growing awareness of the importance of differentiating among different Hispanic populations (e.g., Mexican, Mexican American, Chicano, Puerto Rican, or Cuban), as well as the degree of acculturation (e.g., native or recent immigrant) and available social support (31). Including these types of measures is also important to address inherent biases associated with the current study, in that they are likely attributed to Spanish-language workshop preferences.

Second, additional efforts are needed to understand differences between surface and deeper intervention approaches, which identify language as a defining characteristic as opposed to other intervention strategies that resonate with cultural preferences (32, 33). These elements are especially important for tailoring participant recruitment and delivery efforts, which may be more or less feasible based on the delivery site type and socio-economics of the residents and service area. Further investigations are warranted to better understand program preferences among this population within the context of existing delivery systems and referral patterns. Healthcare settings are already primed to reach diverse populations because of their capacity to use bilingual patient navigators and community health workers (34). This study’s findings suggest that healthcare systems had more capacity to deliver Spanish-language workshops, in contrast to faith-based organizations. Given the traditional importance of religion and church involvement within the Hispanic population (35–38), it may be necessary to increase delivery capacity at faith-based organizations to reach and enroll more Spanish-speaking CDSME program participants.

Third, it will be important to understand how the geography of service delivery affects program utilization. Prior research has revealed a service gap in predominantly Hispanic residential areas, which was combined with a tendency for Hispanic participants to travel further distances to attend CDSMP classes (9). Strategies and partnerships may be needed in certain areas and settings to coordinate transportation for participants without means of travel, which can increase participant retention rates.

Fourth, in future research and practice efforts, it will be important to examine whether Spanish- versus English-language workshops are more effective in terms of achieving positive health and quality of life outcomes among participants. Further investigations should also identify the participant and delivery characteristics associated with greater health benefits received. Some evidence suggests that Hispanic participants in evidence-based programs have greater benefits than White non-Hispanic participants (12, 39), but it is not clear whether these advantages are due to baseline disadvantages of Hispanic participants, or the way the classes are structured or made available in community settings.

## Conclusion

The American Recovery and Reinvestment Act of 2009 (i.e., ARRA) *Communities Putting Prevention to Work: Chronic Disease Self-Management Program* initiative shows the potential for reaching Hispanic participants in a variety of delivery sites. This study provides insight into Spanish-language CDSME program recruitment and utilization with implications for program adoption in underserved Hispanic community settings. To grow the numbers of Hispanic participants reached, it may be important to increase the capacity of communities and organizations to deliver Spanish-language programs and utilize culturally tailored and appropriate recruitment materials and channels.

## Conflict of Interest Statement

The authors declare that the research was conducted in the absence of any commercial or financial relationships that could be construed as a potential conflict of interest.

This paper is included in the Research Topic, “Evidence-Based Programming for Older Adults.” This Research Topic received partial funding from multiple government and private organizations/agencies; however, the views, findings, and conclusions in these articles are those of the authors and do not necessarily represent the official position of these organizations/agencies. All papers published in the Research Topic received peer review from members of the Frontiers in Public Health (Public Health Education and Promotion section) panel of Review Editors. Because this Research Topic represents work closely associated with a nationwide evidence-based movement in the US, many of the authors and/or Review Editors may have worked together previously in some fashion. Review Editors were purposively selected based on their expertise with evaluation and/or evidence-based programming for older adults. Review Editors were independent of named authors on any given article published in this volume.

## References

[1] U.S. Census Bureau. The Next Four Decades: The Older Population in the United States: 2010 to 2050. Washington, DC: U.S. Census Bureau (2010). 138 p.

[2] Humes K Jones N Ramirez R Overview of Race and Hispanic Origin: 2010. Washington, DC: U.S. Department of Commerce Economics and Statistics Administration (2011).

[3] Passel JS Cohn DV US Population Projections: 2005-2050. Washington, DC: Pew Research Center (2008).

[4] United States-México Border Health Commission. Health Disparities and the US-México Border: Challenges and Opportunities. El Paso, TX: United States-México Border Health Commission (2010).

[5] Angel JL Whitfield KE The Health of Aging Hispanics: The Mexican-Origin Population. New York, NY: Springer (2007). 294 p.

[6] Agency for Healthcare Research and Quality. 2013 National Healthcare Disparities Report. Rockville, MD: U.S. Department of Health and Human Services (2014).

[7] Federal Interagency Forum on Aging-Related Statistics. Population: Indicator 1. Number of Older Americans (2012) [cited 2014 July 8]. Available from: http://www.agingstats.gov/Main_Site/Data/2012_Documents/Population.aspx

[8] Administration on Aging. A Statistical Profile of Hispanic Older Americans Aged 65+ (2014) [updated 2010; cited 2014 July 9]. Available from: http://www.aoa.gov/Aging_Statistics/minority_aging/Facts-on-Hispanic-Elderly.aspx

[9] Salazar C Smith ML Perez A Ahn S Ory MG Geospatial characteristics of the chronic disease self-management program: reaching diverse ethnic population in San Antonio, Texas. Tex Public Health Assoc J (2011) 63:16–20.

[10] Livingston G Minushkin S Cohn DV Center PH Hispanics and Health Care in the United States: Access, Information and Knowledge. Washington, DC: Pew Hispanic Center (2008).

[11] Escarce J Kapur K Access to and quality of health care. In: Tienda M Mitchell F, editors. Hispanics and the Future of America. (Chap. 10), Washington, DC: National Academies Press (2006). p. 410–46. 20669436

[12] Smith ML Ahn S Mier N Jiang L Ory MG An evidence-based program to reduce fall-related risk among older adults: a comparison of program efficacy by ethnicity. Calif J Health Promot (2012) 10(1):28–44.

[13] Ory MG Smith ML Kulinski KP Lorig K Zenker W Whitelaw N Self-management at the tipping point: reaching 100,000 Americans with evidence-based programs. J Am Geriatr Soc (2013) 61(5):821–3 10.1111/jgs.12239 23672545

[14] National Council on Aging. Evidence-Based Health Promotion Programs for Older Adults: Key Factors and Strategies Contributing to Program Sustainability. Washington, DC: National Council on Aging (2012).

[15] DuBard CA Gizlice Z. Language spoken and differences in health status, access to care, and receipt of preventive services among US Hispanics. Am J Public Health (2008) 98(11):2021–8. 10.2105/AJPH.2007.119008 18799780 PMC2636430

[16] Pearson WS Ahluwalia IB Ford ES Mokdad AH. Language preference as a predictor of access to and use of healthcare services among Hispanics in the United States. Ethn Dis (2008) 18(1):93–7. 18447107

[17] Cheng EM Chen A Cunningham W. Primary language and receipt of recommended health care among Hispanics in the United States. J Gen Intern Med (2007) 22(Suppl 2):283–8. 10.1007/s11606-007-0346-6 17957412 PMC2078546

[18] Lorig K Living a Healthy Life with Chronic Conditions: Self-Management of Heart Disease, Arthritis, Diabetes, Asthma, Bronchitis, Emphysema & Others. 3rd ed Boulder, CO: Bull Publishing Company (2006). 8, 382 p.

[19] Ory MG Ahn S Jiang L Lorig K Ritter P Laurent DD National study of chronic disease self-management six-month outcome findings. J Aging Health (2013) 25(7):1258–74. 10.1177/0898264313502531 24029414

[20] Ory MG Ahn S Jiang L Smith ML Ritter PL Whitelaw N Successes of a national study of the chronic disease self-management program: meeting the triple aim of health care reform. Med Care (2013) 51(11):992–8. 10.1097/MLR.0b013e3182a95dd1 24113813

[21] Ahn S Basu R Smith ML Jiang L Lorig K Whitelaw N The impact of chronic disease self-management programs: healthcare savings through a community-based intervention. BMC Public Health (2013) 13(1):1141. 10.1186/1471-2458-13-1141 24314032 PMC3878965

[22] Bandura A Social cognitive theory of self-regulation. Organ Behav Hum Decis Process (1991) 50(2):248–87 10.1016/0749-5978(91)90022-L

[23] Kulinski KP Boughtaugh M Smith ML Ory MG Lorig K Setting the stage: measure seletion, coordination, and data collection for a national self-management initiative. Front Public Health (2015) 2:206 10.3389/fpubh.2014.00206 PMC4410349 25964919

[24] U.S. Department of Health and Human Services, Administration on Aging. ARRA – Communities Putting Prevention to Work: Chronic Disease Self-Management Program (2012) [cited 2014 June 14]. Available from: www.cfda.gov/index?s=program&mode=form&tab=step1&id=5469a61f2c5f25cf3984fc3b94051b5f

[25] Stanford School of Medicine. Chronic Disease Self-Management Program (Better Choices, Better Health^®^ Workshop) (2014) [updated 2014; cited 2014 July 12]. Available from: http://patienteducation.stanford.edu/programs/cdsmp.html

[26] United States Department of Agriculture Economic Research Service. Rural-Urban Commuting Area Codes: Overview (2014) [updated 2014; cited 2014 August 7]. Available from: http://www.ers.usda.gov/data-products/rural-urban-commuting-area-codes.aspx#.U-ObPmPqlZU

[27] U.S. Census Bureau. Census 2000, Summary File 1, Table S1702; generated by Scott Horel; using American FactFinder (2012). Available from: http://factfinder2.census.gov

[28] Smith ML Ory MG Ahn S Kulinski KP Jiang L Horel S National dissemination of chronic disease self-management education programs: an incremental examination of delivery characteristics. Front Public Health (2015) 2:227 10.3389/fpubh.2014.00227 PMC4410345 25964923

[29] U.S. Census Bureau. State & County QuickFacts: USA (2014) [updated 2014; cited 2014 August 3]. Available from: http://quickfacts.census.gov/qfd/states/00000.html

[30] U.S. Census Bureau. Hispanic Origin (2012) [cited 2014 August 5]. Available from: http://www.census.gov/population/hispanic/

[31] Gonzalez-Barrera A Lopez MH. A Demographic Portrait of Mexican-Origin Hispanics in the United States. Pew Research Hispanic Trends Project [Internet] (2013). Available from: http://www.pewhispanic.org/2013/05/01/a-demographic-portrait-of-mexican-origin-hispanics-in-the-united-states/

[32] Mier N Ory MG Medina A. Anatomy of culturally sensitive interventions promoting nutrition and exercise in Hispanics: a critical examination of existing literature. Health Promot Pract (2010) 11(4):541–54. 10.1177/1524839908328991 19193933 PMC3780354

[33] Mier N Ory MG Toobert DJ Smith ML Osuna D McKay JR A qualitative case study examining intervention tailoring for minorities. Am J Health Behav (2010) 34(6):822–32. 20604705 PMC3133692

[34] Riddick S Improving access for limited English-speaking consumers: a review of strategies in health care settings. J Health Care Poor Underserved (1998) 9(5):S40–61 10.1353/hpu.2010.0672

[35] Koenig H King D Carson VB Handbook of Religion and Health. 2nd ed New York, NY: Oxford University Press (2012). 1192 p.

[36] Koenig HG Pargament KI Nielsen J. Religious coping and health status in medically ill hospitalized older adults. J Nerv Ment Dis (1998) 186(9):513–21. 10.1097/00005053-199809000-00001 9741556

[37] Musgrave CF Allen CE Allen GJ Spirituality and health for women of color. Am J Public Health (2002) 92(4):557–60 10.2105/AJPH.92.4.557 11919051 PMC1447116

[38] Ahn S Hochhalter AK Moudouni DK Smith ML Ory MG. Self-reported physical and mental health of older adults: the roles of caregiving and resources. Maturitas (2012) 71(1):62–9. 10.1016/j.maturitas.2011.10.011 22137860

[39] Smith ML Cho J Salazar CI Ory MG. Changes in quality of life indicators among chronic disease self-management program participants: an examination by race and ethnicity. Ethn Dis (2013) 23(2):182–8. 23530299

